# Global, regional, and national burden of cutaneous malignant melanoma from 1990 to 2021 and prediction to 2045

**DOI:** 10.3389/fonc.2024.1512942

**Published:** 2024-12-24

**Authors:** Chengling Liu, Xingchen Liu, Li Hu, Xin Li, Haiming Xin, Sailin Zhu

**Affiliations:** ^1^ Center of Burns and Plastic Surgery and Dermatology, The 924th Hospital of Joint Logistics Support Force of the People’s Liberation Army (PLA), Guilin, China; ^2^ Department of Pathology, Changhai Hospital, Naval Medical University, Shanghai, China; ^3^ Department of the Hematology and Oncology, The 924th Hospital of Joint Logistics Support Force of the Chinese People’s Liberation Army (PLA), Guilin, China

**Keywords:** cutaneous malignant melanoma, global burden of disease (GBD), frontier analysis, Bayesian age-period-cohort (BAPC) model, annual percentage changes

## Abstract

**Background:**

Cutaneous Malignant Melanoma (CMM) is a significant global health challenge. Understanding regional differences in CMM prevalence and trends is crucial for developing targeted strategies. To address this, we analyzed epidemiological patterns and investigated risk factors for CMM-related mortality.

**Methods:**

This study analyzed CMM using data from the 2021 Global Burden of Diseases survey, covering 204 countries and territories. We evaluated the number and age-standardized rates of prevalence (ASPR), mortality (ASMR), disability-adjusted life years (ASDR), and annual percentage changes (EAPCs). Trends were stratified by region, country, age, sex, and Sociodemographic Index (SDI). A Bayesian Age-Period-Cohort model projected future prevalence, mortality, and DALYs, while decomposition analysis identified key drivers of CMM burden. Frontier analysis further associated CMM outcomes with socio-demographic development.

**Results:**

In 2021, the global prevalence of CMM reached 833,215 cases, a 161.3% increase since 1990. During this period, the ASPR rose from 19.13 to 25.37 per 100,000, while the ASMR declined from 0.84 to 0.73 per 100,000. DALYs increased by 60.5%, from 1,045,777 to 1,678,836. The high SDI region had the highest ASPR, ASMR, and ASDR. Decomposition analysis identified population growth, demographic aging, and epidemiological changes as equal drivers of CMM DALYs globally. Countries like New Zealand and Australia demonstrated the most significant effective differences, indicating potential for improvement in CMM management. By 2045, the global ASPR is projected to rise to 36.61, with ASMR and ASDR expected to decrease to 0.79 and 10.21 per 100,000.

**Conclusion:**

CMM poses an increasing global health concern, with ASPR steadily rising. While this analysis shows a decline in global ASMR and ASDR overall, these rates are actually increasing in low SDI regions, and projections indicate that this trend will likely continue until 2045.

## 1 Introduction

Cutaneous Malignant Melanoma (CMM) is a common cancer in the Western world with an increasing incidence and continues to be the deadliest form of skin cancer globally, significantly impacting public health (1). Despite advances in understanding and treatment, its incidence has been increasing, highlighting the importance of continued research and intervention efforts (2–4). Understanding CMM’s burden, from its epidemiology to the effectiveness of prevention strategies, is crucial in addressing the disease effectively.

The epidemiology of CMM reveals a complex pattern of incidence and mortality globally. In 2020, there were estimated to be 325,000 new cases and 57,000 deaths due to CMM worldwide. These numbers are projected to increase significantly by 2040, with an estimated 510,000 new cases and 96,000 deaths annually if current trends continue (5). This increase is attributed to various factors, including demographic changes and possibly increasing UV exposure and changes in sun-related behaviors (5).

CMM incidence shows significant regional variation, with the highest rates in Australia and New Zealand (up to 42 per 100,000 annually), compared to much lower rates in Asia and Africa (below 1 per 100,000). This disparity is largely attributable to differences in skin type, UV radiation exposure, and the effectiveness of public health interventions targeting skin cancer prevention (5, 6).

Current epidemiological research is limited by the lack of comprehensive, globally representative data which could provide a more nuanced understanding of CMM across various regions and groups (6, 7). Most studies are concentrated in regions with higher healthcare resources and cancer registry systems, such as North America and Europe (7–9). This limitation hinders the ability to fully understand CMM’s impact in low-resource settings or in populations that historically have not been as deeply studied.

The Global Burden of Disease Study (GBD) offers a thorough framework for comprehending these differences and forecasting CMM patterns in the future (5). This research addresses the gaps in our understanding of CMM from 1990 to 2021, with forecasts to 2045. Moreover, we employed frontier and decomposition analysis to examine the various factors that may contribute to the development of CMM. We aim to investigate trends in incidence and mortality, gender disparities, and the impact of socioeconomic factors on CMM outcomes globally. Our findings intend to inform public health strategies to reduce CMM ‘s burden and contribute to global efforts in combating this disease, promoting more effective prevention, diagnosis, and treatment approaches.

## 2 Methods

### 2.1 Study population and data collection

This cross-sectional study, approved by the 924th Hospital of the Joint Logistics Support Force of the PLA without requiring informed consent, utilized data from the Global Burden of Disease (GBD) 2021 database, accessible at https://vizhub.healthdata.org/gbd-results/. The GBD 2021 study comprehensively assessed health loss across 204 countries and regions, drawing on the latest epidemiological data and refined standardized methods. It identified 371 diseases and injuries as key contributors to health loss. Cutaneous Malignant Melanoma (CMM), characterized within the GBD framework as a malignant skin condition involving abnormal melanocyte proliferation and immune dysfunction, classified under ICD-10: C43, was studied in individuals aged 15 to 95+ years using data from the Global Health Data Exchange (6). The data for this study, covering CMM prevalence, mortality, and disability-adjusted life years (DALYs) from 1990 to 2021, were derived from the GBD 2021 study. The GBD study gathers global health data through a comprehensive methodology that includes systematic literature reviews, hospital records, insurance claims, and national health surveys (10, 11). The GBD uses a rigorous methodology to provide the most accurate global health estimates. It comprehensively accounts for diseases and risks, ensuring measurement comparability across time and regions. Data on CMM prevalence, mortality, and DALYs from 1990 to 2021 were obtained from the GBD Results Tool. The regions included five socialdemographic index (SDI) regions and the global region. The SDI classifies areas into high, high-middle, middle, low-middle, and low categories based on birth rates, income, and education to support health research and policy development (12). We calculated EAPCs via linear regression and complied with STROBE guidelines.

### 2.2 Statistical analysis

In this study, we utilized published estimates of global, regional, and national prevalence, mortality and DALYs for the CMM, disaggregated by sex, location, 5-year age group, and year, based on data from the GBD 2021. DALYs, which reflect both years lost to premature death and years lived with disability owing to a particular health condition, combine years lived with disability and years of life lost to produce a complete assessment of overall health burden. Previous papers provide extensive calculation techniques for age-standardized rates, DALYs, incidence, and prevalence (13, 14). Age-standardized rates (ASRs) for CMM were calculated using the direct method, based on the GBD 2021 world population age standard per 100,000 persons. These included the age-standardized mortality rate (ASMR), prevalence rate (ASPR), and DALY rate (ASDR) (15).

To validate EAPC descriptions, we used Joinpoint analysis to identify global ASR inflection points from 1990 to 2021. Annual percent change (APC) measured percentage changes within specific segments, while average annual percent change (AAPC) assessed average annual ASR changes over time. These tools reinforced our findings by evaluating the statistical significance of ASR trend changes (16).

Decomposition analysis revealed the variables influencing increases in DALYs and worldwide CMM mortality between 1990 and 2021. It evaluated the effects of aging, population increase, and changes in epidemiology. This study’s decomposition analysis was carried out utilizing Gupta’s suggested methodology. We analyzed mortality and DALYs for the global region and five SDI regions. The detailed formulas and methods can be found in previous publications (17).

The Bayesian Age-Period-Cohort (BAPC) model integrates age, period, and cohort effects, allowing for the prediction of illness trends while considering demographic shifts (18). This model was used to forecast CMM prevalence and DALY rates through 2045 for people aged 15 to 95+ globally and across five SDI regions.

We used frontier analysis to explore the relationship between CMM burden and socio-demographic development. This method identified the minimum age-standardized incidence and DALY rates attainable for CMM indicators at a given SDI (19).

In this study, the R software package (version 4.2.3) and jD_GBDR (V2.22, Jingding Medical Technology Co., Ltd.) were used to generate the figures.

## 3 Results

### 3.1 Global burden of cutaneous malignant melanoma

**Figure 1:**
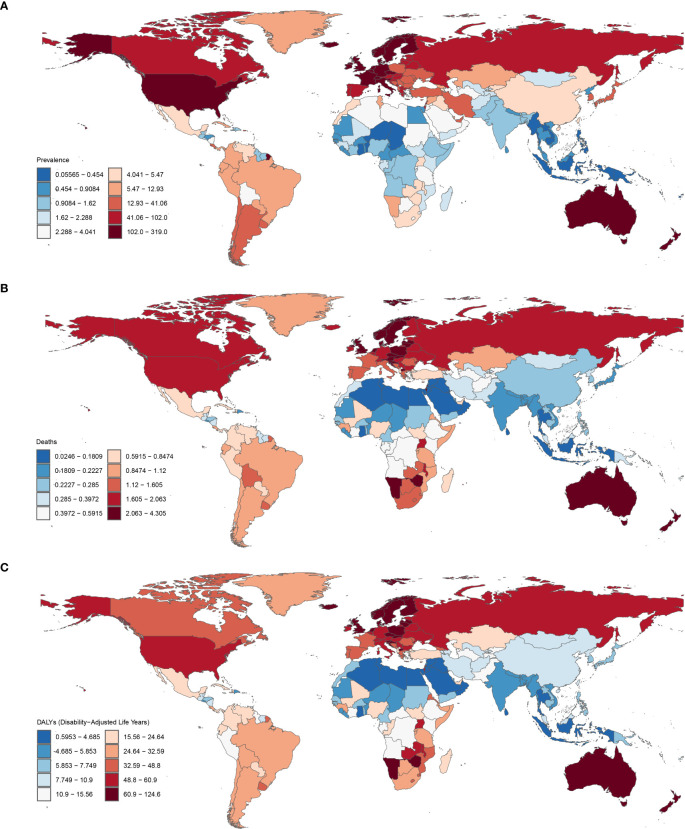
Global ASPR **(A)**, ASMR **(B)** and ASDR **(C)** of CMM in 2021 (per 100,000 population). ASPR, age-standardized prevalence rate; ASMR, age-standardized mortality rate; ASDR, age-standardized disability-adjusted life year rate; CMM, Cutaneous Malignant Melanoma.

The global maps of ASPR, ASMR and ASDR of CMM in 2021 are shown in (**Figures 1A–C**), respectively. Between 1990 and 2021, the global burden of CMM experienced a notable rise in ASPR. However, ASMR and ASDR declined during the same period. In 2021, females had lower ASPR, ASMR and ASDR for CMM compared to males (**Table 1**).

**Table 1:** Age-standardized Prevalence rate, DALYs and Age-standardized Mortality rate of Cutaneous Malignant Melanoma between 1990 to 2021 at the Global and Regional level.

Location	Rate per 100 000 (95% UL)						
	1990			2021			1990-2021
	ASPR	ASDR	ASMR	ASPR	ASDR	ASMR	EAPC^a^ of the ASPR
Global	19.13(18.65-19.55)	24.33(22.39- 25.58)	0.85(0.78-0.89)	25.37(23.98-26.51)	19.63(17.25-21.50)	0.73(0.68-0.73)	1.97(1.60-2.34)
Sex							
Female	19.40(18.75-19.95)	21.01 (18.88-23.18)	0.73(0.66-0.79)	23.65(22.08-25.21)	16.55(13.77-19.21)	0.59(0.51-0.68)	0.72(0.37-1.08)
Male	19.13(18.63-19.54)	28.07(25.29-29.95)	0.99(0.91-1.06)	27.79(26.34-29.26)	23.23(20.32-26.00)	0.90(0.81-0.99)	1.34(0.88-1.80)
SDI							
High	67.15(65.73-68.50)	53.44(51.90-55.21)	1.68(1.62-1.72)	91.36(87.72-94.19)	40.99(38.90-43.36)	1.40(0.68-1.40)	2.07(1.64-2.50)
High middle	11.04(10.59-11.46)	25.51(23.39-28.81)	0.86(0.79-0.90)	24.59(22.03-26.39)	23.27(20.55-25.25)	0.83(0.68-0.83)	3.97(3.68-4.26)
Middle SDl	1.02(0.79-1.15)	10.39(7.95-12.05)	0.37(0.29-0.43)	3.85(2.76-4.59)	10.28(7.68-11.99)	0.38(0.68-0.38)	5.56(5.47-5.64)
Low middle	0.47(0.34-0.58)	7.29(5.36-9.23)	0.25(0.19-0.32)	1.27(0.89-1.56)	8.55(5.94-10.86)	0.29(0.68-0.29)	4.10(3.93-4.27)
Low SDI	0.75(0.47-0.96)	14.78(9.49-18.73)	0.49(0.32-0.62)	1.29(0.77-1.74)	14.60(8.78-19.29)	0.48(0.68-0.48)	2.01(1.74-2.28)
Regions							
Andean Latin America	1.37(1.07-1.76)	20.28(15.39-26.71)	0.81(0.63-1.05)	5.57(4.13-7.53)	19.99(15.07-25.54)	0.82(0.62-1.04)	6.40(6.18-6.62)
Australasia	222.85(210.88-236.40)	196.05(184.77-207.02)	6.52(6.11-6.96)	266.83(244.68-289.56)	109.97(100.62-120.58)	3.88(3.48-4.26)	1.74(1.17-2.32)
Caribbean	2.38(2.55-2.23)	12.12(10.67-14.53)	0.44(0.39-0.51)	5.71(4.99-6.50)	13.90(11.61-17.08)	0.50(0.43-0.59)	3.44(3.18-3.69)
Central Asia	3.55(3.87-3.20)	19.59(17.94-21.44)	0.74(0.67-0.81)	4.72(4.10-5.43)	12.54(8.08-18.22)	0.60(0.52-0.67)	1.49(0.91-2.08)
Central Europe	16.58(18.08-15.70)	52.52(49.63-56.73)	1.72(1.62-1.86)	51.87(45.99-57.30)	58.34(52.78-63.49)	2.10(1.92-2.27)	5.03(4.66-5.41)
Central Latin America	1.51(1.56-1.46)	13.55(13.18-13.91)	0.51(0.49-0.53)	6.20(5.50-6.93)	18.18(16.32-20.06)	0.69(0.62-0.76)	5.94(5.72-6.16)
Central Sub-Saharan Africa	0.59(0.92-0.42)	12.95(9.34-19.85)	0.48(0.34-0.71)	1.05(0.70-1.79)	13.86(9.12-21.99)	0.52(0.33-0.79)	2.19(1.74-2.65)
East Asia	0.67(0.84-0.44)	9.02(5.86-11.31)	0.31(0.21-0.40)	4.06(2.17-5.49)	7.90(4.41-10.44)	0.28(0.15-0.36)	8.09(7.82-8.35)
Eastern Europe	13.74(14.70-13.12)	37.68(36.11-39.96)	1.14(1.09-1.21)	42.16(38.68-45.59)	53.97(49.21-59.18)	1.74(1.59-1.89)	4.52(4.24-4.80)
Eastern Sub-Saharan Africa	1.40(1.76-0.90)	27.02(17.77-33.92)	0.89(0.59-1.12)	2.42(1.36-3.60)	27.03(15.59-38.06)	0.88(0.51-1.21)	2.11(1.79-2.44)
High-income Asia Pacific	6.25(6.65-5.85)	6.91(6.27-7.47)	0.24(0.22-0.25)	14.98(12.50-16.70)	6.91(5.76-7.68)	0.23(0.19-0.25)	4.25(3.76-4.74)
High-income North America	132.8(135.4-129.3)	78.81(76.10-81.65)	2.32(2.23-2.37)	133.27(127.26-137.47)	51.06(48.23-54.14)	1.74(1.62-1.81)	0.87(0.44-1.29)
North Africa and Middle East	2.32(3.25-1.20)	8.97(4.47-13.12)	0.36(0.18-0.50)	11.41(6.11-14.23)	7.60(3.97-9.30)	0.30(0.15-0.37)	5.93(5.76-6.10)
Oceania	2.32(3.25-1.20)	8.05(5.48-14.27)	0.33(0.23-0.58)	0.28(0.20-0.44)	7.92(5.48-12.68)	0.32(0.22-0.50)	0.73(0.59-0.87)
South Asia	0.27(0.47-0.19)	5.78(4.11-7.78)	0.19(0.14-0.25)	1.00(0.63-1.43)	6.43(4.28-8.93)	0.21(0.14-0.29)	4.37(4.09-4.65)
Southeast Asia	0.34(0.45-0.23)	4.88(3.75-6.95)	0.17(0.14-0.25)	1.00(0.63-1.43)	5.35(3.70-7.07)	0.20(0.14-0.27)	3.05(2.91-3.18)
Southern Latin America	0.34(0.46-0.23)	25.75(24.39-26.97)	0.89(0.84-0.93)	0.42(0.29-0.57)	29.91(28.10-32.00)	1.07(0.99-1.14)	4.59(4.08-5.10)
Southern Sub-Saharan Africa	0.22(0.31-0.17)	30.53(22.44-41.35)	1.11(0.76-1.45)	4.81(2.80-6.72)	37.61(22.57-47.44)	1.38(0.79-1.69)	2.96(2.65-3.28)
Tropical Latin America	5.22(5.53-4.92)	26.99(26.12-27.94)	0.92(0.87-0.95)	8.31(7.94-8.70)	26.99(25.58-28.13)	0.98(0.91-1.03)	4.22(4.02-4.41)
Western Europe	2.59(3.61-1.89)	53.37(51.83-54.90)	1.68(1.62-1.72)	113.04(108.22-117.23)	52.59(49.66-55.65)	1.74(1.61-1.82)	3.66(3.18-4.13)
Western Sub-Saharan Africa	3.13(3.26-3.00)	12.26(6.13-16.92)	0.42(0.22-0.57)	1.03(0.36-1.51)	12.22(5.06-16.92)	0.43(0.19-0.57)	1.82(1.57-2.06)

EAPC, estimated annual percentage change: SDl, Sociodemographic Index; Ul, uncertainty interval; DALY, disability-adjusted life-years

a EAPC is expressed as 95% Cls.

CMM prevalence increased from 833,215.66 (95% UI: 813,312.99–849,960.71) in 1990 to 2,177,566.14 (95% UI: 2,057,878.68–2,274,067.92) in 2021, representing a 161.3% rise. Additionally, the ASPR rose from 19.13 (95% UI: 18.65–19.55) in 1990 to 25.37 (95% UI: 23.98–26.51) in 2021, reflecting a 32.6% increase. The number of CMM incidence cases increased from 124,319.84 (95% UI: 119,603.87–127,610.46) in 1990 to 303,104.61 (95% UI: 281,717.64–318,904.82) in 2021, marking a 143.8% rise. Furthermore, ASIR increased from 2.98 (95% UI: 2.87–3.06) in 1990 to 3.56 (95% UI: 3.31–3.75) in 2021, indicating a 19.3% growth. The CMM mortality increased from 33,060.98 (95% UI: 30,546.44–34,529.54) in 1990 to 61,549.73 (95% UI: 54,852.45–66,265.02) in 2021, representing an 86.2% rise. However, the ASMR decreased from 0.84 (95% UI: 0.78–0.89) in 1990 to 0.73 (95% UI: 0.65–0.79) in 2021, indicating a 13.8% decline. On the other hand, DALYs increased from 1,045,777.53 (95% UI: 959,373.17–1,103,849.01) in 1990 to 1,678,836.31 (95%UI: 1,474,533.65–1,837,368.79) in 2021, reflecting a 60.5% rise. Meanwhile, the ASDR for DALYs decreased from 24.33 (95% UI: 22.39–25.58) in 1990 to 19.63 (95% UI: 17.25–21.50) in 2021, marking a 13.9% reduction.

### 3.2 SDI regional burden of CMM

**Figure 2:**
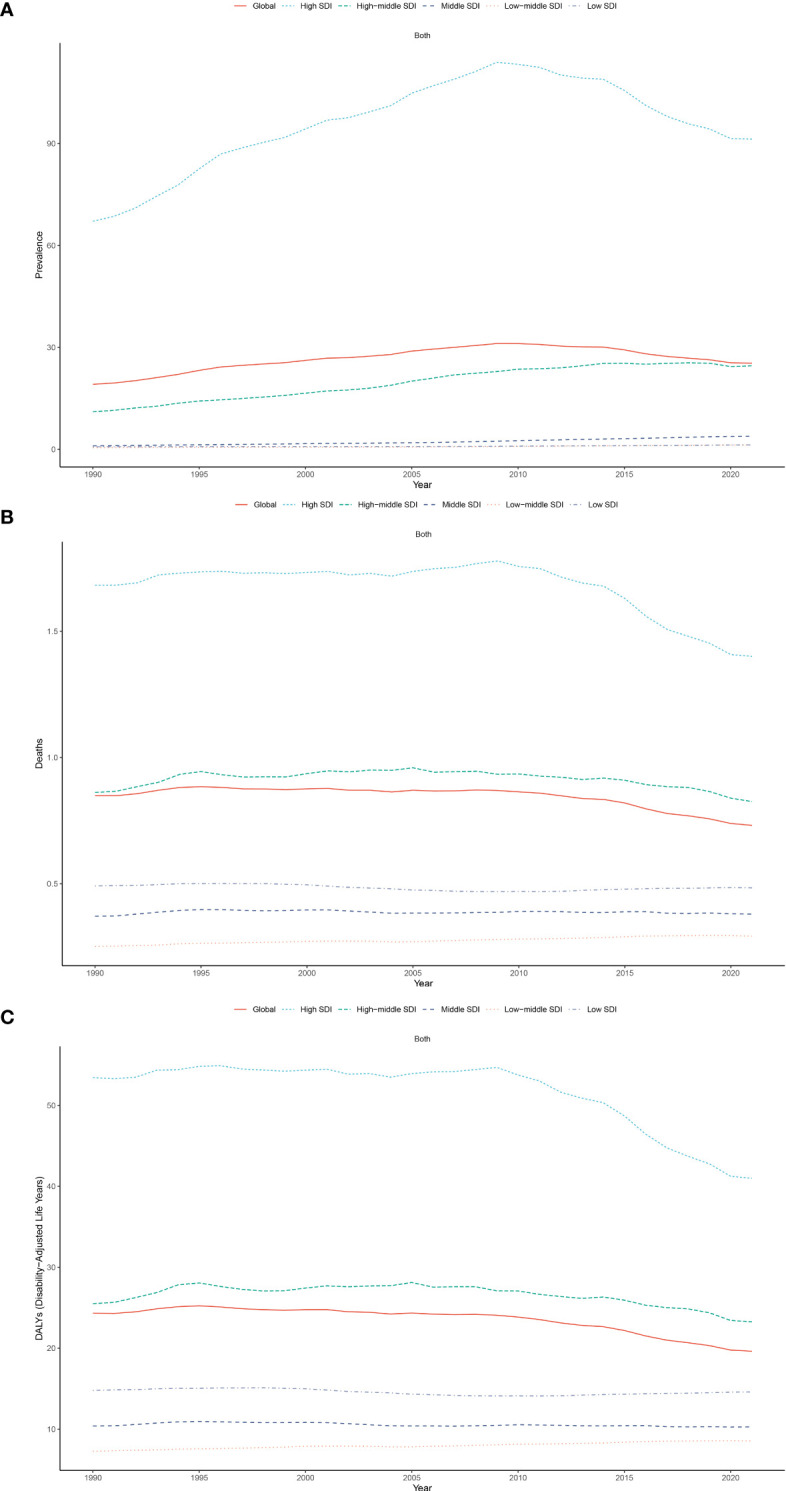
Trends in ASPR **(A)**, ASMR **(B)**, and ASDR **(C)** for CMM across five Sociodemographic Index (SDI) regions from 1990 to 2021.

**Figure 3:**
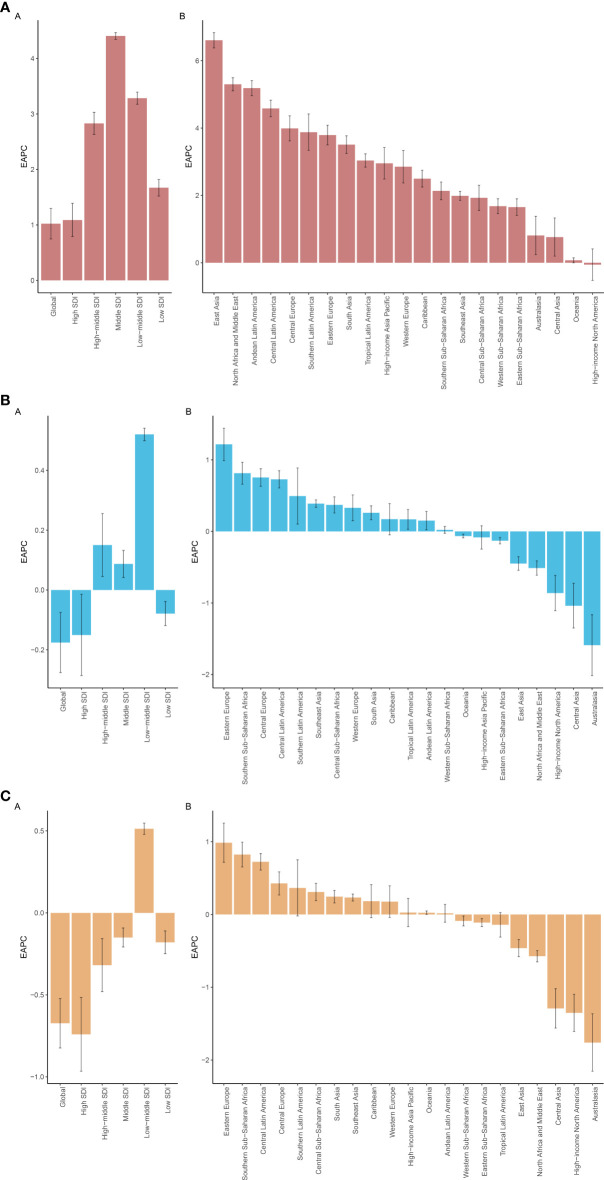
ASPR **(A)**, ASMR **(B)**, and ASDR **(C)** for CMM across different geographical regions in 2021.

Between 1990 and 2021, the ASPR burden of CMM increased across all SDI regions. The most significant rise occurred in the middle SDI region, with an EAPC of 4.41 (95% UI: 4.35–4.47) per 100,000 populations. In contrast, the ASDR showed a slight decline across all SDI regions, except for the low-middle region, which experienced a minor increase with an EAPC of 0.51 (95% UI: 0.48–0.55). Additionally, the ASMR rose in three regions, with the largest increase observed in the low-middle region, reaching 0.52 (95% UI: 0.50–0.54) (**Figures 2A-C**; **3A-C**).

In 2021, the high SDI region consistently recorded the highest ASPR, ASMR and ASDR for CMM (ASPR of 91.36, ASMR of 1.40, and ASDR of 40.99 per 100,000 population), whereas the low-middle SDI region had the lowest (**Table 1**). These figures substantially exceeded those in the low SDI region, where ASIR was 72.36, ASPR was 478, and DALYs rate was 19.73 per 100,000 populations (**Table 1**)

### 3.3 Geographic regional burden of CMM

In 2021, Australasia recorded the highest number of CMM cases, with a prevalence of 816,674.79 (95% UI: 776,086.87–844,343.72). Australasia also had the highest ASPR at 266.83, the highest ASMR at 3.88, and the highest ASDR at 109.97 per 100,000 people. In contrast, Oceania had the lowest ASPR at 0.28, while Southeast Asia recorded both the lowest ASDR at 5.35 and the lowest ASMR at 0.20 per 100,000 people.

**Figure 4:**
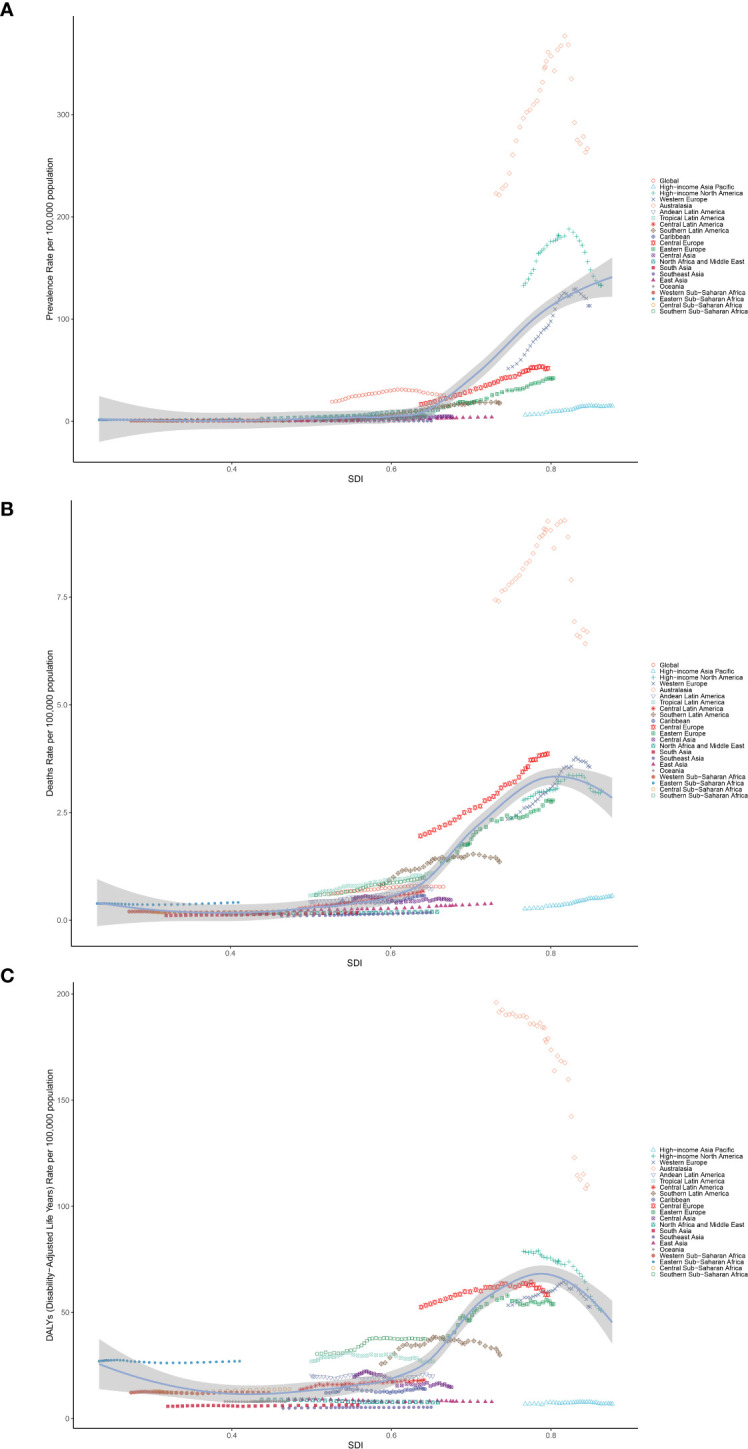
The EAPCs for ASPR **(A)**, ASMR **(B)**, and ASDR **(C)** due to CMM from 1990 to 2021, for both sexes, across GBD regions and SDI quintiles. DALYs, disabilityadjusted life-years; EAPC, estimated annual percentage change; GBD, Global Burden of Disease; SDI, Socio-demographic Index.

Across the GBD regions from 1990 to 2021, the ASPR of CMM increased in all regions, with Eastern Asia experiencing the most significant rise (EAPC = 8.09) and Oceania showing the smallest increase (EAPC = 0.73). Regarding ASMR, it rose in thirteen GBD regions but declined in eight, with Central Latin America seeing the largest increase (EAPC = 2.94) and Central Asia the most substantial decrease (EAPC = -0.43). Similarly, ASDR also increased in thirteen regions and declined in eight, with Central Latin America experiencing the most significant rise (EAPC = 2.49) and Australasia recording the largest decline (EAPC = -0.81) (**Figures 3A-C**; **4A-C**).

### 3.4 National burden of CMM

In 2021, the United States recorded the highest number of CMM cases, reaching 90,444.95. New Zealand, however, reported the highest ASPR at 461.33 per 100,000 individuals, as well as the highest ASDR at 179.36 per 100,000. Between 1990 and 2021, the ASPR for CMM increased across most countries, with the exception of the Kyrgyz Republic, which saw a slight decrease (EAPC = -0.34). The Kingdom of Saudi Arabia experienced the most significant rise in ASPR, with an EAPC of 8.64 (**Figure 1A**). A similar trend was observed in the ASMR, which rose in 177 countries, with the Republic of Mauritius showing the largest increase (EAPC = 6.58) (**Figure 1B**). Additionally, the ASDR rose in 172 countries, again with the Republic of Mauritius reporting the highest increase (EAPC = 7.61).

### 3.5 Age-specific and sex burden of CMM in the elderly

**Figure 5:**
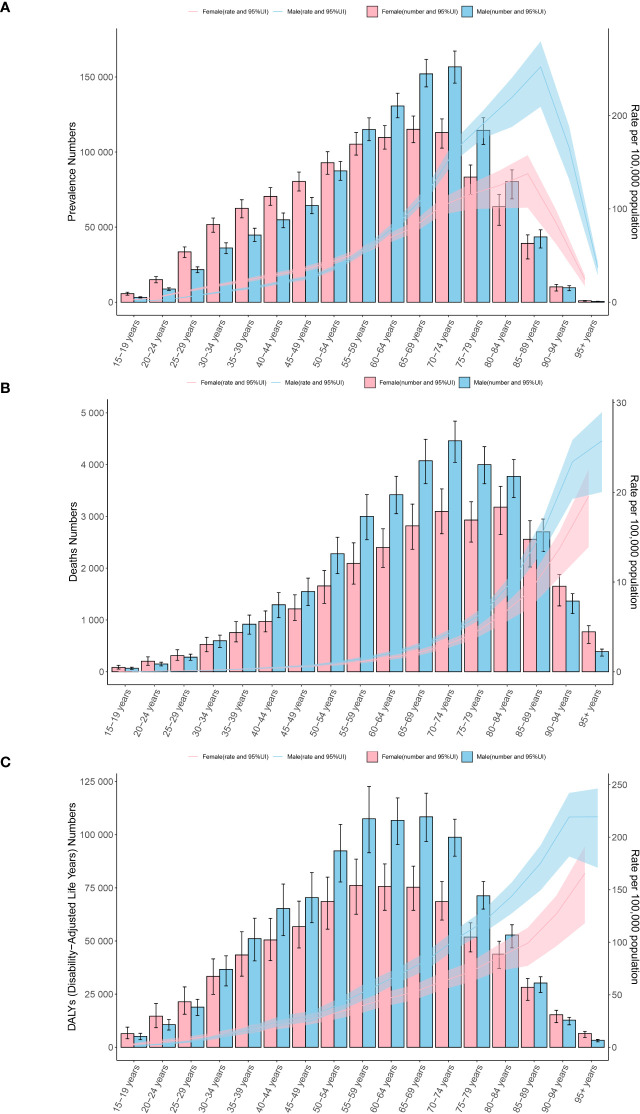
Age-specific patterns by sex for ASPR **(A)**, ASMR **(B)**, and ASDR **(C)** associated with CMM at the global level in 2021. Error bars indicate the 95% uncertainty interval (UI) for the number of cases. Shading indicates the 95% UI for the rates. DALYs, disability-adjusted life-years.

In 2021, the incidence of CMM was primarily concentrated in the 70 to 74-year age group. Among individuals aged over 55, the ASIR was higher in males than in females. A similar pattern was observed in the ASPR and ASMR. With increasing age, the ASIR, ASPR, ASMR, and ASDR all followed an upward trend. Notably, the ASDR reached its highest point in the 95+ age group, while the ASPR peaked in the 85 to 89 age group. The ASMR also peaked in the 95+ age group, and the ASIR reached its maximum in the 90 to 94 age group, with males consistently showing higher rates than females across these metrics (**Figures 5A-C**).

### 3.6 BAPC analysis

**Figure 6:**
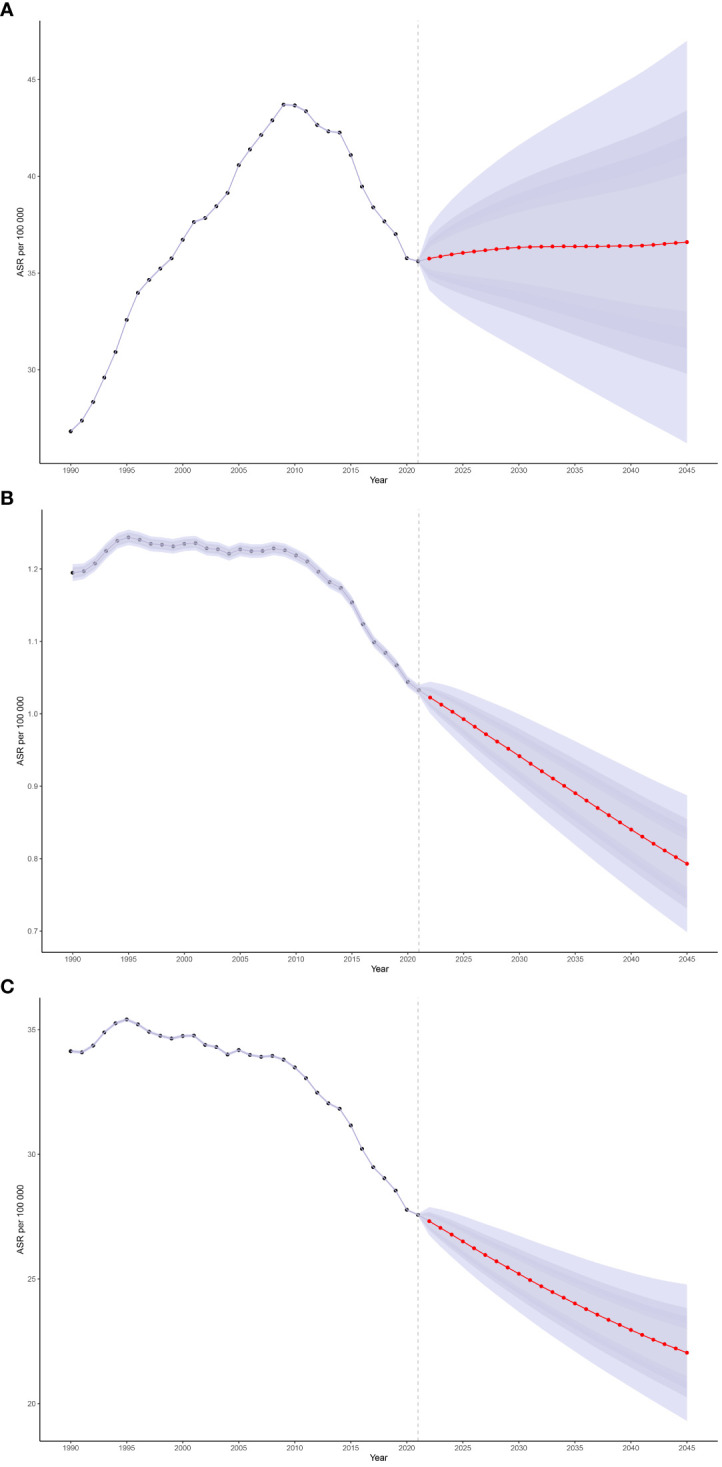
The global trends in ASPR **(A)**, ASMR **(B)**, and ASDR **(C)** from 2021 to 2045 for CMM were predicted using Bayesian age-period-cohort (BAPC) models.

To analyze post-2021 trends in the ASPR, ASMR, and ASDR of CMM, we applied Bayesian age-period-cohort (BAPC) models to forecast these rates globally from 2021 to 2045. Projections indicate a global increase in the ASPR, rising from 35.62 to 36.61 per 100,000 by 2045 (**Figure 6A**). In contrast, the ASMR is expected to decrease slightly from 1.03 in 2021 to 0.79 in 2045, and the ASDR from 19.70 to 10.21 over the same period (**Figure 6B**). Furthermore, the ASPR is projected to increase across most age groups, except for those aged 65 to 79. The highest ASPR by 2045 is predicted to be 248.13 per 100,000 among individuals aged 85 to 89 (**Figure 6C**).

### 3.7 Decomposition analysis

**Figure 7:**
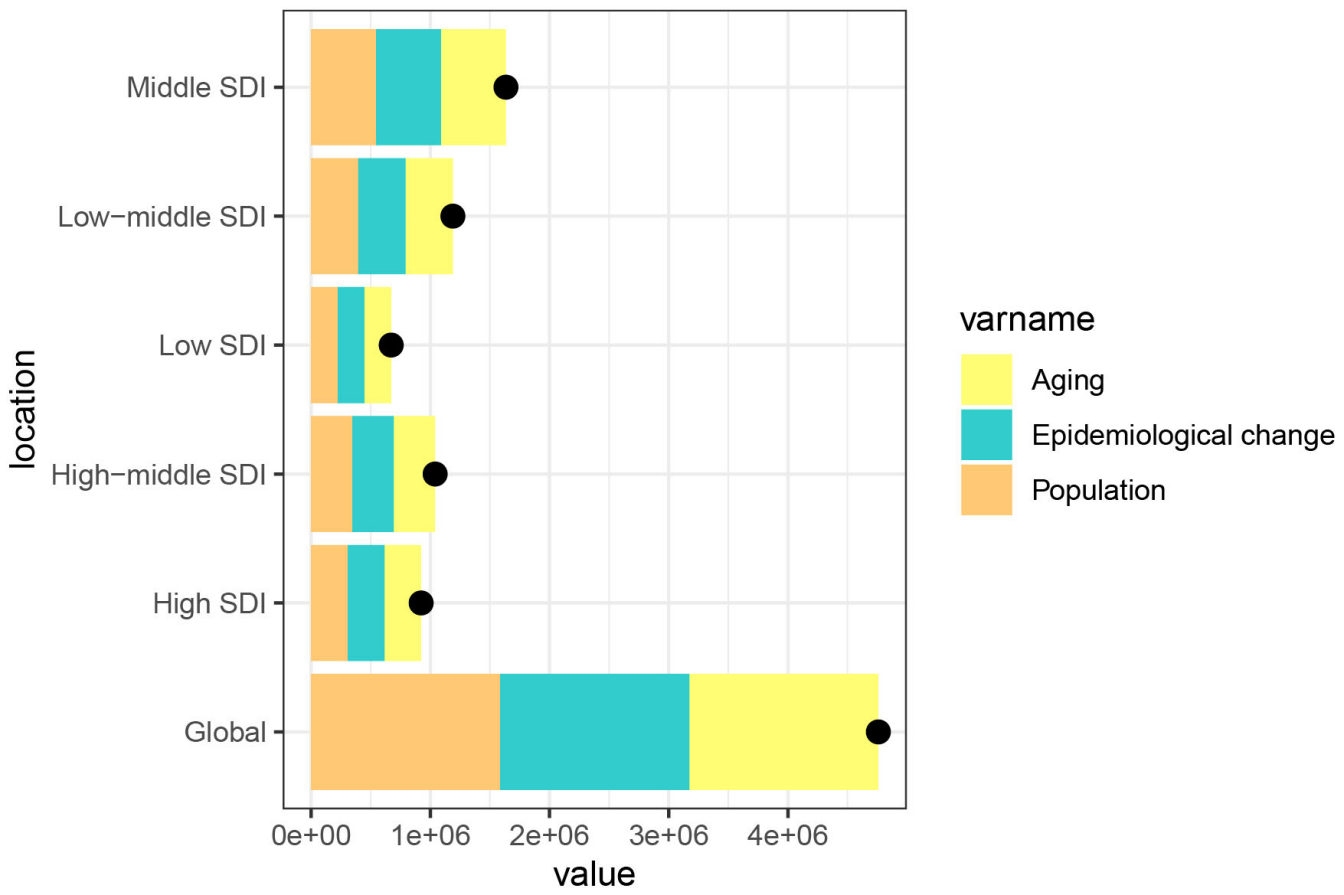
Changes in the DALYs numbers of CMM according to the three causes from 1990 to 2021 at the global level and by SDI quintile and WHO regions. The black dot represents the overall value of incidence change contributed from all causes. DALY, disability-adjusted life year; CMM, Cutaneous Malignant Melanoma; WHO, World Health Organization.

We conducted a decomposition analysis to assess global DALY trends and those within five SDI regions, focusing on the roles of population growth, demographic aging, and epidemiological changings. The global DALY rate significantly increased, with the middle SDI region seeing the highest rise, amounting to 1,634,782.94. Globally, population growth, aging, and epidemiological changes equally contributed to the 33.33% increase in DALY burden from 1990 to 2019, a pattern mirrored across all SDI regions (**Figure 7**).

### 3.8 Frontier analysis in DALY rate of CMM

**Figure 8:**
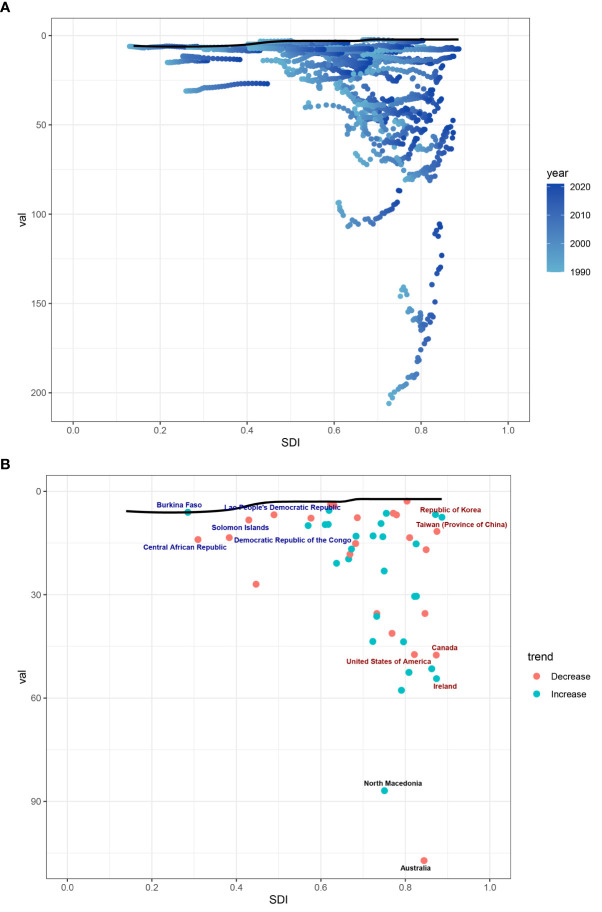
**(A)** Frontier analysis of CMM based on SDI and ASDR from 1990 to 2021. The color scale represents the years from 1990, depicted in black, to 2021, shown in blue. The frontier is delineated in a solid black color. **(B)** Frontier analysis based on SDI and CMM ASDR in 2021. The frontier line is black; countries and territories are represented as dots. The top 15 countries with the most considerable effective difference of ASDR from the frontier line are marked in black words; Red dots indicate an increase in ASDR of CMM from 1990 to 2021; blue dots indicate a decrease in ASDR of CMM between 1990 and 2021.

To better understand trends in DALY rates associated with CMM, we conducted a frontier analysis across 204 countries and regions from 1990 to 2021, using ASDR as a key metric. Countries were categorized along a frontier line based on their SDI levels, with the frontier representing those with the lowest DALY rates relative to their SDI. The “effective difference” reflects the gap between a nation’s actual and achievable DALYs, which can be reduced by optimizing sociodemographic resources. Notably, New Zealand, Australia, and North Macedonia had the largest effective differences, indicating significantly higher ASDR compared to similarly developed nations. In contrast, Burkina Faso, Lao PDR, and the Republic of Korea had the lowest ASDR relative to their development levels (**Figures 8A, B**).

## 4 Discussion

Cutaneous Malignant Melanoma (CMM) is one of the most dangerous types of skin cancer and remains a significant public health concern worldwide (20). CMM originates from melanocytes, the pigment-producing cells in the skin, and its incidence has been rising steadily over the past decades. CMM is a multifactorial disease influenced by both genetic and environmental factors. Excessive ultraviolet (UV) radiation exposure, particularly UVB, is a primary environmental risk factor that leads to DNA damage in melanocytes (21). On the genetic side, mutations in genes such as BRAF and NRAS have been implicated in the pathogenesis of melanoma (22). Additionally, immune dysfunction plays a critical role in the disease progression, with immune checkpoint pathways becoming key targets in modern CMM therapies. Immunotherapy has revolutionized CMM treatment, particularly through the use of immune checkpoint inhibitors (ICIs). ICIs, such as programmed death-1 (PD-1) inhibitors and cytotoxic T-lymphocyte-associated antigen-4 (CTLA-4) inhibitors, have significantly improved survival outcomes. For example, pembrolizumab, a PD-1 inhibitor, achieved a 5-year overall survival rate of 41% in patients with advanced CMM, with approximately 25% experiencing complete remission. This marks a transformative shift in the therapeutic landscape for advanced melanoma (23, 24). However, immunotherapies’ advantages are not shared fairly as their accessibility is still unequal around the world. These innovative therapies are widely available in developed nations, but comparable access is hampered in poor nations by a lack of funding, high costs, and inadequate infrastructure (25). Hence, early detection and prevention remain the most effective strategies in reducing CMM mortality.

Our study aligns with previous research on the global burden of CMM, particularly regarding incidence trends. It is well established that regions like Australia and New Zealand have the highest incidence rates, largely driven by factors such as high UV exposure, specific skin types, and the presence of robust national cancer registries (26, 27). Our findings reaffirm this, as Australasia continues to lead in both incidence, DALYs, and mortality rates.

A unique contribution of our study is the detailed focus on low- and middle-income regions (low-SDI and middle-SDI regions), which have been relatively underexplored in prior research. While most previous studies have concentrated on high-income countries due to better access to cancer registries and healthcare systems (6, 8, 28), our analysis reveals a contrasting trend. Mortality rates in high-SDI regions have declined, but low- and middle-SDI regions have seen increases in both incidence and mortality, highlighting an urgent need for improved cancer management strategies in these areas.

We also employed decomposition analysis to identify the key drivers of changes in the global DALYs related to CMM from 1990 to 2021. Our findings indicate that population growth, demographic aging, and epidemiological changes are the primary factors behind the global increase in CMM -related DALYs. Notably, our study offers a deeper focus on regional disparities, identifying low- and middle-SDI countries as disproportionately affected by these demographic changes. This level of detail provides a richer understanding of healthcare inequalities than many similar studies, which often emphasize high-income regions.

In our frontier analysis, we assessed how efficiently countries manage their CMM burden relative to their socio-demographic development. Countries such as New Zealand, Australia, and North Macedonia exhibited the largest “effective differences,” indicating that their CMM burden was higher than expected despite having advanced healthcare systems. This suggests that these countries could benefit from further improvements(improved primary prevention and changing lifestyles) in CMM management (20). On the other hand, countries like Burkina Faso, Lao PDR, sand the Republic of Korea showed the smallest differences, indicating that they manage their CMM burden efficiently despite limited resources. These findings suggest that underperforming countries may require targeted interventions, including UV protection strategies, while efficient low-SDI countries could serve as models for resource optimization and cost-effective healthcare management (26). This analysis offers valuable insights for policy changes and international collaborations aimed at improving CMM outcomes, particularly in low- and middle-SDI regions.

Our study utilized data from 204 countries and regions, providing one of the most geographically comprehensive analyses to date. This approach revealed not only the stark contrasts between high- and low-SDI regions but also highlighted previously underreported disparities within regions. Using the BAPC model, we forecast future trends in CMM prevalence, mortality, and DALYs, providing critical insights for long-term public health planning. Our projections indicate that while mortality may continue to decline in high-SDI regions, the global burden of CMM will remain significant, with prevalence expected to rise by 2045. These advanced methods gave us a clearer understanding of the factors driving the CMM burden, including demographic shifts, epidemiological changes, and regional disparities in healthcare systems. Such insights are crucial for guiding policymakers in developing interventions that align with the specific socio-demographic conditions of different regions.

Additionally, our study observed gender disparities, with males aged over 55 exhibiting higher mortality and morbidity rates. This suggests the need for gender-specific public health strategies, including tailored health messaging and screening campaigns to address men’s particular risk behaviors and healthcare access challenges.

Finally, the rising CMM burden in low-SDI regions, combined with limited healthcare infrastructure, underscores the necessity of international collaboration and funding. Such efforts are vital for improving early detection, diagnosis, and treatment in these regions.

Despite its strengths, this study has certain limitations. First, the reliance on data from the GBD study means that the accuracy of our findings is contingent on the quality of the cancer registries in each country. In many low- and middle-income countries, underreporting and incomplete data collection could lead to an underestimation of the true burden of CMM.

Second, while our predictive models offer valuable insights, they are based on current trends and do not account for potential breakthroughs in CMM treatment, changes in sun exposure behaviors, or new public health interventions that could alter future incidence and mortality rates. As a result, the long-term projections should be interpreted with caution.

Lastly, our analysis did not consider certain region-specific factors, such as cultural behaviors and environmental differences, which could affect CMM risk. Future research could focus on exploring these variables more thoroughly to provide a more nuanced understanding of CMM risk across diverse populations.

## 5 Conclusion

This study provides a comprehensive analysis of the global burden of CMM from 1990 to 2021, highlighting key trends in incidence, mortality, and DALYs. Our findings confirm the persistently high CMM incidence in regions like Australasia and New Zealand, while also revealing increasing rates in low- and middle-SDI regions, where healthcare infrastructure is limited. Through decomposition analysis, we identified population growth, demographic aging, and epidemiological shifts as major drivers of the global CMM burden. The frontier analysis further demonstrated disparities in CMM management efficiency across countries, with some high-SDI regions underperforming and certain low-SDI regions achieving better-than-expected outcomes. These insights underscore the need for targeted interventions, gender-specific strategies, and international collaboration to reduce the growing CMM burden and optimize healthcare resource allocation globally.

## Data Availability

The original contributions presented in the study are included in the article/supplementary material. Further inquiries can be directed to the corresponding authors.

## References

[1] Schadendorf D Fisher DE Garbe C Gershenwald JE Grob JJ Halpern A. Melanoma. Nat Rev Dis Primers. (2015) 1:15003. doi: 10.1038/nrdp.2015.3 27188223

[2] Nwabudike LC Oproiu AM Dogaru IM Costache M Onisor C Tatu AL. Therapy delayed is therapy denied: A case report of melanoma misdiagnosed as diabetic foot ulcer. Clin Cosmet Investig Dermatol. (2021) 14:1909–12. doi: 10.2147/CCID.S337545 PMC8720861 35002272

[3] Bujoreanu FC Bezman L Radaschin DS Niculet E Bobeica C Craescu M. Nevi, biologics for psoriasis and the risk for skin cancer: A real concern? (Case presentation and short review). Exp Ther Med. (2021) 22:1354. doi: 10.3892/etm.2021.10789 34659500 PMC8515562

[4] Secinti IE Gursoy D Erturk T Dede I Ozgur T Dogan E. Should we report Breslow density, a new concept in cutaneous melanoma. Malays J Pathol. (2021) 43:397–404. 34958061

[5] Arnold M Singh D Laversanne M Vignat J Vaccarella S Meheus F. Global burden of cutaneous melanoma in 2020 and projections to 2040. JAMA Dermatol. (2022) 158:495–503. doi: 10.1001/jamadermatol.2022.0160 35353115 PMC8968696

[6] De Pinto G Mignozzi S La Vecchia C Levi F Negri E Santucci C. Global trends in cutaneous Malignant melanoma incidence and mortality. Melanoma Res. (2024) 34:265–75. doi: 10.1097/CMR.0000000000000959 PMC11045545 38391175

[7] Huang J Chan SC Ko S Lok V Zhang L Lin X. Global incidence, mortality, risk factors and trends of melanoma: A systematic analysis of registries. Am J Clin Dermatol. (2023) 24:965–75. doi: 10.1007/s40257-023-00795-3 37296344

[8] MacKie RM Hauschild A Eggermont AM. Epidemiology of invasive cutaneous melanoma. Ann Oncol. (2009) 20 Suppl 6:vi1–7. doi: 10.1093/annonc/mdp252 PMC2712590 19617292

[9] Rastrelli M Tropea S Rossi CR Alaibac M. Melanoma: epidemiology, risk factors, pathogenesis, diagnosis and classification. In Vivo. (2014) 28:1005–11. 25398793

[10] Kuang Z Wang J Liu K Wu J Ge Y Zhu G. Global, regional, and national burden of tracheal, bronchus, and lung cancer and its risk factors from 1990 to 2021: findings from the global burden of disease study 2021. EClinicalMedicine. (2024) 75:102804. doi: 10.1016/j.eclinm.2024.102804 39290907 PMC11406099

[11] Zhang K Kan C Han F Zhang J Ding C Guo Z. Global, regional, and national epidemiology of diabetes in children from 1990 to 2019. JAMA Pediatr. (2023) 177:837–46. doi: 10.1001/jamapediatrics.2023.2029 PMC10318549 37399036

[12] Pu Y He L Wang X Zhang Y Zhao S Fan J. Global, regional, and national levels and trends in burden of urticaria: A systematic analysis for the Global Burden of Disease study 2019. J Glob Health. (2024) 14:04095. doi: 10.7189/jogh.14.04095 38818613 PMC11140429

[13] Zhang Y Dong S Ma Y Mou Y. Burden of psoriasis in young adults worldwide from the global burden of disease study 2019. Front Endocrinol (Lausanne). (2024) 15:1308822. doi: 10.3389/fendo.2024.1308822 38414821 PMC10897041

[14] Wang R Li Z Liu S Zhang D. Global, regional and national burden of inflammatory bowel disease in 204 countries and territories from 1990 to 2019: a systematic analysis based on the Global Burden of Disease Study 2019. BMJ Open. (2023) 13:e065186. doi: 10.1136/bmjopen-2022-065186 PMC10069527 36977543

[15] Lv B Lan JX Si YF Ren YF Li MY Guo FF. Epidemiological trends of subarachnoid hemorrhage at global, regional, and national level: a trend analysis study from 1990 to 2021. Mil Med Res. (2024) 11:46. doi: 10.1186/s40779-024-00551-6 38992778 PMC11241879

[16] Yang K Yang X Jin C Ding S Liu T Ma B. Global burden of type 1 diabetes in adults aged 65 years and older, 1990-2019: population based study. BMJ. (2024) 385:e078432. doi: 10.1136/bmj-2023-078432 38866425 PMC11167563

[17] Ruan R Liu X Zhang Y Tang M He B Zhang QW. Global, regional, and national advances toward the management of rheumatic heart disease based on the global burden of disease study 2019. J Am Heart Assoc. (2023) 12:e028921. doi: 10.1161/JAHA.122.028921 37366108 PMC10356074

[18] Wang K Zhao Y Cao X. Global burden and future trends in psoriasis epidemiology: insights from the global burden of disease study 2019 and predictions to 2030. Arch Dermatol Res. (2024) 316:114. doi: 10.1007/s00403-024-02846-z 38530431

[19] Xie Y Bowe B Mokdad AH Xian H Yan Y Li T. Analysis of the Global Burden of Disease study highlights the global, regional, and national trends of chronic kidney disease epidemiology from 1990 to 2016. Kidney Int. (2018) 94:567–81. doi: 10.1016/j.kint.2018.04.011 30078514

[20] Whiteman DC Green AC Olsen CM. The growing burden of invasive melanoma: projections of incidence rates and numbers of new cases in six susceptible populations through 2031. J Invest Dermatol. (2016) 136:1161–71. doi: 10.1016/j.jid.2016.01.035 26902923

[21] Gandini S Autier P Boniol M. Reviews on sun exposure and artificial light and melanoma. Prog Biophys Mol Biol. (2011) 107:362–6. doi: 10.1016/j.pbiomolbio.2011.09.011 21958910

[22] Shain AH Yeh I Kovalyshyn I Sriharan A Talevich E Gagnon A. The genetic evolution of melanoma from precursor lesions. N Engl J Med. (2015) 373:1926–36. doi: 10.1056/NEJMoa1502583 26559571

[23] Cancer Genome Atlas Network. Genomic classification of cutaneous melanoma. Cell. (2015) 161:1681–96. doi: 10.1016/j.cell.2015.05.044 PMC4580370 26091043

[24] Hamid O Robert C Daud A Hodi FS Hwu WJ Kefford R. Five-year survival outcomes for patients with advanced melanoma treated with pembrolizumab in KEYNOTE-001. Ann Oncol. (2019) 30:582–8. doi: 10.1093/annonc/mdz011 PMC6503622 30715153

[25] Ralli M Botticelli A Visconti IC Angeletti D Fiore M Marchetti P. Immunotherapy in the treatment of metastatic melanoma: current knowledge and future directions. J Immunol Res. (2020) 2020:9235638. doi: 10.1155/2020/9235638 32671117 PMC7338969

[26] SChadendorf D van Akkooi A Berking C Griewank KG Gutzmer R Hauschild A. Melanoma. Lancet. (2018) 392:971–84. doi: 10.1016/S0140-6736(18)31559-9 30238891

[27] Chen ST Geller AC Tsao H. Update on the epidemiology of melanoma. Curr Dermatol Rep. (2013) 2:24–34. doi: 10.1007/s13671-012-0035-5 23580930 PMC3619431

[28] Apalla Z Lallas A Sotiriou E Lazaridou E Ioannides D. Epidemiological trends in skin cancer. Dermatol Pract Concept. (2017) 7:1–6. doi: 10.5826/dpc.0702a01 PMC5424654 28515985

